# Supplementary figures and images for: Exploring bacterial key genes and therapeutic agents for breast cancer among the Ghanaian female population: Insights from In Silico analyses
Source: PLoS One. 2024 Nov 25;19(11):e0312493. doi: 10.1371/journal.pone.0312493 (PMC11588272; doi:10.1371/journal.pone.0312493)

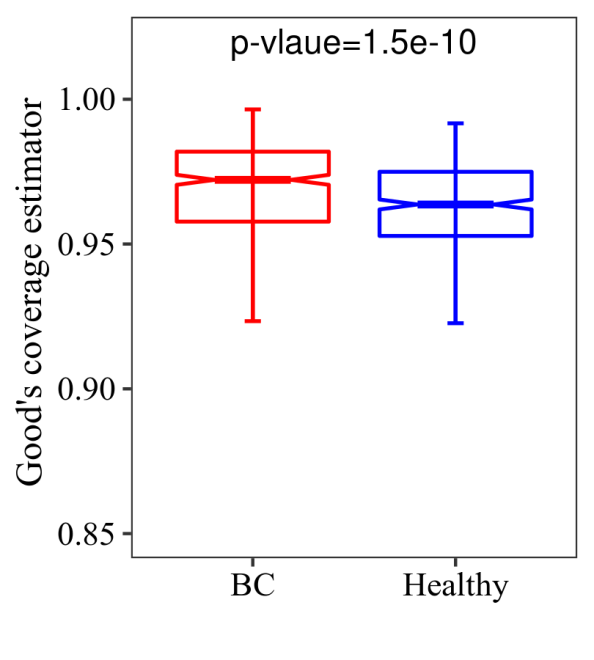


S1 Fig. Good’s coverage estimates between BC and healthy control.

Supplement: S1 Fig — (DOCX) [file pone.0312493.s001.docx]
