# Supplementary material for: Exploring bacterial key genes and therapeutic agents for breast cancer among the Ghanaian female population: Insights from In Silico analyses
Source: PLoS One. 2024 Nov 25;19(11):e0312493. doi: 10.1371/journal.pone.0312493 (PMC11588272; doi:10.1371/journal.pone.0312493)
Supplement: S1 Table — (DOCX) [file pone.0312493.s002.docx]

S1 Table: Comparison of different microbial communities alpha diversity indices with a significant difference between breast BC (n = 520) and healthy patients (n = 442) based on Wilcoxon-Mann-Whitney test and Cliff’s Delta.

| **Alpha Diversity** | **Disease Status** | **Wilcoxon-Mann-Whitney test** | |  | **Cliff's Delta** | |
| --- | --- | --- | --- | --- | --- | --- |
|  |  | **Median**  **(IQR)** | **P-value** |  | **Mean**  **(SD)** | **Effect size** |
| Observed | Breast  cancer | 210  (152 , 277) | 4.678e-11 |  | 214.514  (80.636) | 0.522 (large) |
|  | Healthy | 247  (199.5, 300) |  |  | 249.031  (69.464) |  |
| Chao1 | Breast  cancer | 496.625  (342.38, 716.60) | 7.873e-13 |  | 530.734  (244.751) | 0.752 (large) |
|  | Healthy | 632.279  (474.38, 793.36) |  |  | 644.777  (222.425) |  |
| ACE | Breast  cancer | 540.62  (364.97, 755.39) | 1.061e-11 |  | 565.420  (261.441) | 0.621 (large) |
|  | Healthy | 669.09  (512.77, 829.25) |  |  | 680.057  (231.731) |  |
| Good  coverage | Breast  cancer | 0.972  (0.957, 0.981) | 1.506e-10 |  | 0.968  (0.018) | -0.246 (small) |
|  | Healthy | 0.963  (0.952, 0.974) |  |  | 0.961  (0.017) |  |
