# Supplementary material for: Exploring bacterial key genes and therapeutic agents for breast cancer among the Ghanaian female population: Insights from In Silico analyses
Source: PLoS One. 2024 Nov 25;19(11):e0312493. doi: 10.1371/journal.pone.0312493 (PMC11588272; doi:10.1371/journal.pone.0312493)
Supplement: S2 Table — (DOCX) [file pone.0312493.s003.docx]

S2 Table: Summary of bKGs corresponding to our identified metabolic pathways which are significantly more abundant in BC patients group.

| **Pathway Name** | **Genes** |
| --- | --- |
| sucrose degradation III (sucrose invertase) | Ams, hxk1, hxk2, glk1, pgi1, gpi1, cinv2 |
| peptidoglycan maturation (meso-diaminopimelate containing) | ftsW, mrdB, mtgA, pbpC, mrcA, mrdA, ftsI, mrcB, yfeW, dacB, ampH, dacC, dacA, dacD, ldtD, mepH, mepS, ldtE |
| superpathway of guanosine nucleotides de novo biosynthesis II | guaB, guaA, gmk, nrdE, nrdF, nrdA, nrdB, ndk, adk, nrdD |
| pentose phosphate pathway | tktB, gnd, rpiB, tkt, tal, rpiA, zwf rpe, talB,  tktA |
| chondroitin sulfate degradation I (bacterial) | cslA, ugl |
| superpathway of purine nucleotides de novo biosynthesis II | adk, gmk, guaA, guaB, ndk, nrdA, nrdB, nrdD, nrdE, nrdF, purA, purB, purE, purF, purH, purK, purL, purM, purT |
| superpathway of pyrimidine ribonucleosides salvage | cdd, udk, cmk, adk, ndk, udk, rihA, rihC, rihB, upp, pyrH, ndk, adk, pyrG |
| superpathway of purine nucleotides de novo biosynthesis I | agk1, ndpk1, rnr2a, rnr1, aak6, pura |
| glycolysis I (from glucose 6-phosphate) | ptsG, crr, bglF, manZ, manY, manX, pgi, kduI, pfkA, pfkB, fbp, glpX, yggF, ybhA, fbaB, fbaA, tpiA, gapA, pgk, gpmA, gpmM, eno, pykA, pykF, ppsA |
| pyruvate fermentation to propanoate I | frdA, frdB, frdC, frdD, fumA, fumB, fumC, fumD, fumE, maeA, mdh, scpA, scpC |
| glycolysis II (from fructose 6-phosphate) | pfkA, pfkB, fbp, glpX, yggF, ybhA, phoA, fbaB, fbaA, tpiA, gapA, pgk, gpmA, gpmM, eno, pykA, pykF, ppsA |
| TCA cycle VI (obligate autotrophs) | Ppc, mqo, gltA, acnA, acnB, acnA, acnB, icd, fumC, fumD, fumE, fumB, fumA |
