# Supplementary material for: Exploring bacterial key genes and therapeutic agents for breast cancer among the Ghanaian female population: Insights from In Silico analyses
Source: PLoS One. 2024 Nov 25;19(11):e0312493. doi: 10.1371/journal.pone.0312493 (PMC11588272; doi:10.1371/journal.pone.0312493)
Supplement: S3 Table — (DOCX) [file pone.0312493.s004.docx]

S3 Table: Distribution of identified top 10 bKGs corresponding to their metabolic pathways.

| **Pathway** | **Gene name** |
| --- | --- |
| pyruvate fermentation to propanoate I | mdh |
| glycolysis I (from glucose 6-phosphate) | pykF, gapA, pgi, tpiA, pgk, pfkA, ppsA, pykA |
| glycolysis II (from fructose 6-phosphate) | pykF, gapA, tpiA, pgk, pfkA, ppsA, pykA |
| pentose phosphate pathway | zwf, |
