# Supplementary material for: Exploring bacterial key genes and therapeutic agents for breast cancer among the Ghanaian female population: Insights from In Silico analyses
Source: PLoS One. 2024 Nov 25;19(11):e0312493. doi: 10.1371/journal.pone.0312493 (PMC11588272; doi:10.1371/journal.pone.0312493)
Supplement: S4 Table — (DOCX) [file pone.0312493.s005.docx]

S4 Table: Metadata of host-protein of breast cancer patients obtained by reviewing published articles were used in this study

| **Articles** | **Hub protein** | **Drug** |
| --- | --- | --- |
| Wu et al. 2020 (1) | BUB1B, CDC45, MAD2L1, PLK1, CDC20, AURKA | NA |
| Zhao et al. 2019 (2) | KRAS, CDH2, VEGFA, ZEB2, NTRK2, TWIST1 | NA |
| Wang et al. 2020 (3) | CCNE1, CHEK1, PLK1, CENPN, DSCC1, FAM64A, UBE2T, UBE2C | NA |
| Wu et al. 2020 (4) | FOXA1, GATA3, ESR1, PGR, CCND1, AR, TFF1, GREB1, NRIP1, KRT18 | NA |
| Chen et al. 2020 (5) | RPL4, HSP90AA1, SRC, ESR1, PPP2CA, HSPA8, ACTB | NA |
| Zhou et al. 2020 (6) | CXCL8, CD44, BMP7, MMP9 | NA |
| Yang et al. 2019 (7) | TPX2, CCNB2, BUB1, CENPE, KIF2C, NDC80, TOP2A, MELK, KIF20A, CKS2 | NA |
| Liu et al. 2021 (8) | ITGB1, FERMT2, ITGA5, CEMIP, HGF, MCAM, TGFBR1, F2RL2 | NA |
| Lv et al. 2019 (9) | CDC20, CCNA2, CCNB2, TTK, CENPF, BUB1, CENPA, CENPE | NA |
| Chuan et al. 2020 (10) | CXCR4, CXCL10 | NA |
| Jin et al. 2018 (11) | CCNB1, AURKA, BIRC5, BUB1B, ZWINT, CCNB2, CDK1, CDKN3, CENPF, PTTG1, TOP2A, CDC20, TPX2, UBE2C, PRC1 | NA |
| Tian et al. 2018 (12) | GGTA1P, CRABP2, SYNPO2, NAP1L2, MKI67, COL4A6, MAGI2-AS3 | NA |
| He et al. 2015 (13) | MYEOV2, DUSP1, UQCRQ | NA |
| Shi et al. 2020 (14) | BUB1B, CCNB2, AURKA, CDK1, CDT1, KIF20A, KIF2C, HJURP, KIF4A, MELK, UBE2C, TPX2 | NA |
| Li et al. 2018 (15) | PLK1, AURKA, BIRC5, BUB1, CCNB1, BUB1B, CDK1, MAD2L1, NDC80, KIF11 | NA |
| Zhang et al. 2020 (16) | ASPM, CDC20, AURKA, TOP2A, NUSAP1, KIF20A, CEP55, CDK1, CCNB2, UBE2C | NA |
| Cao et al. 2021 (17) | HSPB1, IFI16, TPX2 | NA |
| Liu et al. 2020 (18) | CDC20, CCNA2, CDK1, CCNB1, CCNB2, BUB1B, CDCA8, BUB1, KIF11, TOP2A | NA |
| Zhang et al. 2020 (19) | HSPD1, HSPH1, TXN, ATIC | NA |
| Wei et al. 2021 (20) | CDK1, TOP2A, CCNB1, MKI67, TTK, BUB1, PLK1, CCNA2 | NA |
| Dong et al. 2018 (21) | AR, SOX8, RAB30, C9orf152, NRK | NA |
| Wu et al. 2012 (22) | MCM3, BCL2, MCM7, TGFB2 | NA |
| Yang et al. 2018 (23) | NOS3, BDNF | NA |
| He et al. 2021 (24) | TGFBR3, C1QB, CEP55, SPP1, IFI6, KIAA0101, PBK, DCN, FZD7, KRT5, HIST1H2BO, SPAG5 | NA |
| Amjad et al. 2020 (25) | FOS, SMC4, FN1, KIF11, RACGAP1, JUN | NA |
| Zhu et al. 2020 (26) | CXCL12, CXCL12, FOS | NA |
| Zhai et al. 2020 (27) | NUF2, FAM83D | NA |
| Li et al. 2021 (28) | CD3G, CD3D, CD3E, GRAP2, FYN, ITK | NA |
| Xiao et al. 2021 (29) | NCAPG, CCNB1, MCM4, RRM2 | NA |
| Lu et al. 2019 (30) | ACACB, PHLPP1, UBC, TGFB1, ACTB | NA |
| Wang et al. 2019 (31) | MELK, CDK1, TOP2A, CCNB1, KIF11, CCNA2 | NA |
| Yan et al. 2021 (32) | FN1, DDR2, EFNA3, RHOQ, NRP1 | NA |
| Peng et al. 2020 (33) | TP53, SMURF1, BTRC | NA |
| Lin et al. 2020 (34) | TOP2A, CCNB1, RAC1, KIF20A, NUSAP1, RRM2, ASPM, BIRC5, CEP55, BUB1B | NA |
| Zhou and Yang 2020 (35) | IL1β, PTGS2, CXCL8 | NA |
| Lv et al. 2020 (36) | IL6, PTPRC, SELL, SPN, CD40 | NA |
| Bai et al. 2020 (37) | STAT3, STAT1, STAT6, STAT2, XBP1, BCL2L1, ESCO2, CYB5D2 | NA |
| Hao et al. 2020 (38) | RRM2, CDC20, CCNB2, BUB1B, CDK1, CCNA2 | Drug-Set-1 |
| Dong et al. 2018 (39) | JUN, EGFR, IGF1, ESR1 | Drug-Set-2 |
| Wang et al. 2019 (40) | SIDT1, GPR160, ANKRD30A, CA12 | NA |
| Pei et al. 2020 (41) | CCNB2, TPX2, EXO1, CENPA, SKA1, FOXM1, PLK1, SGO1, RAD54L, KIF4A, CDC20 | NA |
| Li et al. 2020 (42) | TTK, CCNE1, EXO1 | NA |
| Cai et al. 2019 (43) | KIF4A, TPX2, CDCA8, CCNA2, BUB1B | NA |
| Takeshita et al. 2020 (44) | CTNNB1, TP53, EGFR, ERBB2, HSPB1 | NA |
| Tang et al. 2019 (45) | VCAM1, ANLN, BUB1, SKA3, JUN, NUSAP1, CKAP2L, TTK, FOS, STAT5A | NA |
| Qi et al. 2019 (46) | TOP2A, MAD2L1, PIK3R1, FEN1, EPRS, EXO1, BCL2, MCM4, PTTG1, PSMD14, CDKN3, RRM2, H2AFZ, CCNE2, FGF2, EGFR | NA |
| Wang et al. 2018 (47) | JUN, ATF3, STAT1, FN1, FOS, COL1A1 | NA |
| Fang and Zhang 2017 (48) | RPS14, RPS9, RPL11, RPL10A | NA |
| Lou et al. 2018 (49) | PRDM10, MAPK1 | NA |
| Fu et al. 2019 (50) | KIF18B, CASC5, CKAP2L, KIF23, SKA1, CDCA5, MCM6, FAM83D, GINS1 | NA |
| Bao et al. 2019 (51) | BCL11A, FAM171A1, FOXC1, RGMA | NA |
| Zhong et al. 2020 (52) | CT83, FABP7, ART3, TTYH1 | NA |
| Bhar et al. 2013 (53) | CCL2, NFIB, BRD4, CD47, HPGD, NPC1L1, CSNK1E, PTEN, ADAM9, PTPN2 | NA |
| Wang et al. 2018 (54) | TOP2A, BIRC5, NDC80, CDK1, CCNB1 | NA |
| Dashti et al. 2020 (55) | CCNA2, MAD2L1, RAD51-AS1, LINC01089 | NA |
| Peng et al. 2017 (56) | TP53, GAPDH, PCNA, CCND1, HRAS | NA |
| Liu et al. 2015 (57) | FN1, FOS, IL6 | NA |
| Bao et al. 2020 (58) | AURKB, GINS2, UHRF1, POLE2, SPC24, E2F2, MCM10 | NA |
| Yin et al. 2021 (59) | PARD6B, GRM4, SSTR2, PRR15, COX6C | NA |
| Zhang et al. 2019 (60) | UBA5, CREB1, ARF3, SIAH1, KLHL3, TRIM69, HECTD1, MMP9, MEX3C, UBE2Q2, FBXO22, ASB6, EIF4A3, PXN | NA |
| Zheng et al. 2017 (61) | TP53INP1, SPI1, CAPG, LEF1, PBX3, PLAGL1, EGFR, TCF7L2, SYK | NA |
| Wang et al. 2018 (62) | MAPK14, NOTCH1 | NA |
| Wang et al. 2020 (63) | TOP2B, ACLY, SRSF1, EFTUD2, DDX5, CCT2, DHX9 | NA |
| Zeng et al. 2018 (64) | FGF2, GSK3B, RAC1, ERBB2, PIK3R1, PXN, RAC2, HSP90AA1 | NA |
| Qin and Chen 2016 (65) | FSTL3, TUBA1C, DUSP8, KLF6, EIF3B, KIF20A, PTPRK, UBR2, ZSCAN20, DEPDC1, AURKA, UNG | NA |
| Zhang et al. 2017 (66) | FN1, CD44, NGF, SERPINE1, CCNA2 | NA |
| Yuan et al. 2019 (67) | FN1, EGFR, PTPRC, JAK3, TUBB3 | NA |
| Hong et al. 2020 (68) | CCNB2, CDC20, PTTG1, CCNB1, BUB1B, CCNE2, TTK | NA |
| Alam et al. 2022 (69) | BUB1, ASPM, TTK, CCNA2, CENPF, RFC4, and CCNB1 | Drug-Set-3 |
| Alam et al. 2022 (70) | AKR1C1, IRF9, OAS1, OAS3, SLCO2A1, NT5E, NQO1, ANGPT1, FN1, ATF6B, HPGD, BCL11A, and TP53INP1 | Drug-Set-4 |
| Alam et al. 2022 (71) | TOP2A, BIRC5, AURKB, ACTB, ASPM, and BUB1B | Drug-Set-5 |
| Alam et al. 2022 (72) | EGFR, FN1, EZH2, MET, CDK1, AURKA, TOP2A, and BIRC5 | Drug-Set-6 |
| Rodrigues et al. 2022 (73) | CREB3L1, HLA, CYP2B6, CYP2C19, CYP3A4, ABCB1, PI3KR1, RAPTOR, CYP2D6, CYP19A1, CSMD1, CYP19A1, LPHN2, ROBO1, SNTG1, PIK3CA, and GRIK1 | Drug-Set-7 |
| Balbuena-Rebolledo et al. 2021 (74) | EGFR/HER2 | Drug-Set-8 |
| Pathak et al. 2022 (75) | PS1 | Drug-Set-9 |
| Jubie et al. 2021 (76) | HER2 | Drug-Set-10 |
| Khanjani et al. 2021 (77) | HER2 | Drug-Set-11 |
| Song et al. 2021 (78) | FGFR2, KCNN4 | Drug-Set-12 |
| Detroja et al. 2022 (79) | RPSA, SEC61A1, ITGB1, PSMB5, PSME3, SNRNP70, SRSF3 | Drug-Set-13 |
| Firoozbakht et al. 2022 (80) | NA | Drug-Set-14 |
| Rui et al. 2022 (81) | RBPJ | Drug-Set-15 |
| Mok et al. 2021 (82) | HCN2 and HCN3 | Drug-Set-16 |
| Al-Taie et al. 2021 (83) | NA | Drug-Set-17 |
| Brasó-Maristany et al. 2021 (84) | HER2, ERBB2 | NA |
| Li et al. 2022 (85) | DLAT, SLC31A1, ATP7A and ATP7B | NA |
| Ren et al. 2022 (86) | CFH, GAS6, MME AND OGN | NA |
| Griguolo et al. 2022 (87) | HER2 | NA |
| Prat et al. 2022 (88) | HER2DX, HER2 | NA |
| Cebulski et al. 2022 (89) | FNDC5 | NA |
| Meng et al. 2022 (90) | RanBP9 | NA |
| Zhu et al. 2022 (91) | PROX1 | NA |
| Fang et al. 2022 (92) | VDAC1 | NA |
| Panagopoulou et al. 2022 (93) | ENPP2 | NA |
| Yin et al. 2022 (94) | KRT13 | NA |
| Regua et al. 2022 (95) | ABI1 | NA |
| Grassini et al. 2022 (96) | HER2 | NA |
| Liu et al. 2022 (97) | SETDB1 | NA |
| Yuan et al. 2022 (98) | AURKB | NA |
| Chen et al. 2022 (99) | BUB1, CCNA2, and PACC1 | NA |
| Shi et al. 2022 (100) | RRM2 | NA |
| **Top-ranked HubGs (At least in 7 articles)** | TOP2A, CCNB1, BUB1B, CCNA2, FN1, BUB1, CDC20, EGFR, CDK1, AURKA | 528 |

**References**

1. Wu J, Lv Q, Huang H, Zhu M, Meng D. Screening and identification of key biomarkers in inflammatory breast cancer through integrated bioinformatic analyses. Genet Test Mol Biomarkers. 2020;24(8):484–91.

2. Zhao CH, Qu L, Zhang H, Qu R. Identification of breast cancer-related circRNAs by analysis of microarray and RNA-sequencing data: An observational study. Medicine (Baltimore). 2019;98(46):e18042.

3. Wang Y, Zhu M, Guo F, Song Y, Fan X, Qin G. Identification of Tumor Microenvironment-Related Prognostic Biomarkers in Luminal Breast Cancer. Front Genet. 2020;11(November).

4. Wu JR, Zhao Y, Zhou XP, Qin X. Estrogen receptor 1 and progesterone receptor are distinct biomarkers and prognostic factors in estrogen receptor-positive breast cancer: Evidence from a bioinformatic analysis. Biomed Pharmacother. 2020;121(June 2019):109647.

5. Chen J, Liu C, Cen J, Liang T, Xue J, Zeng H, et al. KEGG-expressed genes and pathways in triple negative breast cancer: Protocol for a systematic review and data mining. Medicine (Baltimore). 2020;99(18):e19986.

6. Zhou Z, Wu B, Tang X, Ke R, Zou Q. Comprehensive analysis of fibroblast growth factor receptor (FGFR) family genes in breast cancer by integrating online databases and bioinformatics. Med Sci Monit. 2020;26.

7. Yang K, Gao J, Luo M. Identification of key pathways and hub genes in basal-like breast cancer using bioinformatics analysis. Onco Targets Ther. 2019;12:1319–31.

8. Liu S, Song A, Wu Y, Yao S, Wang M, Niu T, et al. Analysis of genomics and immune infiltration patterns of epithelial-mesenchymal transition related to metastatic breast cancer to bone. Transl Oncol. 2021;14(2):100993.

9. Lv X, He M, Zhao Y, Zhang L, Zhu W, Jiang L, et al. Identification of potential key genes and pathways predicting pathogenesis and prognosis for triple-negative breast cancer. Cancer Cell Int. 2019;19(1):1–12.

10. Chuan T, Li T, Yi C. Identification of CXCR4 and CXCL10 as potential predictive biomarkers in triple negative breast cancer (TNBC). Med Sci Monit. 2020;26:1–11.

11. Jin H, Huang X, Shao K, Li G, Wang J. Integrated Analysis Revealed Hub Genes in Breast Cancer. 2018;

12. Tian T, Gong Z, Wang M, Hao R, Lin S, Liu K, et al. Identification of long non-coding RNA signatures in triple-negative breast cancer. Cancer Cell Int. 2018;18(1):1–10.

13. He J, Yang J, Chen W, Wu H, Yuan Z, Wang K, et al. Molecular features of triple negative breast cancer: Microarray evidence and further integrated analysis. PLoS One. 2015;10(6).

14. Shi G, Shen Z, Liu Y, Yin W. Identifying Biomarkers to Predict the Progression and Prognosis of Breast Cancer by Weighted Gene Co-expression Network Analysis. Front Genet. 2020;11(December):1–12.

15. Li MX, Jin LT, Wang TJ, Feng YJ, Pan CP, Zhao DM, et al. Identification of potential core genes in triple negative breast cancer using bioinformatics analysis. Onco Targets Ther. 2018;11:4105–12.

16. Zhang X, Yang L, Chen W, Kong M. Identification of Potential Hub Genes and Therapeutic Drugs in Malignant Pleural Mesothelioma by Integrated Bioinformatics Analysis. 2020;250022(4):656–70.

17. Cao W, Jiang Y, Ji X, Guan X, Lin Q, Ma L. Identification of novel prognostic genes of triple-negative breast cancer using meta-analysis and weighted gene co-expressed network analysis. Ann Transl Med. 2021;9(3):205–205.

18. Liu S, Liu X, Wu J, Zhou W, Ni M, Meng Z, et al. Identification of candidate biomarkers correlated with the pathogenesis and prognosis of breast cancer via integrated bioinformatics analysis. Medicine (Baltimore). 2020;99(49):e23153.

19. Zhang K, Jiang K, Hong R, Xu F, Xia W, Qin G, et al. Identification and characterization of critical genes associated with tamoxifen resistance in breast cancer. PeerJ. 2020;8:1–16.

20. Wei LM, Li XY, Wang ZM, Wang YK, Yao G, Fan JH, et al. Identification of hub genes in triple-negative breast cancer by integrated bioinformatics analysis. Gland Surg. 2021;10(1):799–806.

21. Dong P, Yu B, Pan L, Tian X, Liu F. Identification of Key Genes and Pathways in Triple-Negative Breast Cancer by Integrated Bioinformatics Analysis. 2018;2018.

22. Wu JZ, Lu P, Liu R, Yang TJ. Transcription regulation network analysis of MCF7 breast cancer cells exposed to estradiol. Asian Pacific J Cancer Prev. 2012;13(8):3681–5.

23. Yang M, Li H, Li Y, Ruan Y, Quan C. Identification of genes and pathways associated with MDR in MCF-7/MDR breast cancer cells by RNA-seq analysis. Mol Med Rep. 2018;17(5):6211–26.

24. He Y, Cao Y, Wang X, Jisiguleng W, Tao M, Liu J, et al. Identification of Hub Genes to Regulate Breast Cancer Spinal Metastases by Bioinformatics Analyses. 2021;2021.

25. Amjad E, Asnaashari S, Sokouti B, Dastmalchi S. Systems biology comprehensive analysis on breast cancer for identification of key gene modules and genes associated with TNM-based clinical stages. Sci Rep. 2020;10(1):1–14.

26. Zhu C, Ge C, He J, Zhang X, Feng G, Fan S. Identification of Key Genes and Pathways Associated With Irradiation in Breast Cancer Tissue and Breast Cancer Cell Lines. Dose-Response. 2020;18(2):1–8.

27. Zhai X, Yang Z, Liu X, Dong Z, Zhou D. Identification of NUF2 and FAM83D as potential biomarkers in triple-negative breast cancer. PeerJ. 2020;8:1–16.

28. Li L, Huang H, Zhu M, Wu J. Identification of hub genes and pathways of triple negative breast cancer by expression profiles analysis. Cancer Manag Res. 2021;13:2095–104.

29. Xiao X, Zhang Z, Luo R, Peng R, Sun Y, Wang J, et al. Identification of potential oncogenes in triple_negative breast cancer based on bioinformatics analyses. Oncol Lett. 2021;21(5):1–11.

30. Lu X, Gao C, Liu C, Zhuang J, Su P, Li H, et al. Identification of the key pathways and genes involved in HER2-positive breast cancer with brain metastasis. Pathol Res Pract. 2019;215(8):152475.

31. Wang G. Identification of Potential Crucial Genes and Key Pathways in Breast Cancer Using Bioinformatic Analysis. 2019;10(August):1–17.

32. Yan LR, Wang A, Lv Z, Yuan Y, Xu Q. Mitochondria-related core genes and TF-miRNA-hub mrDEGs network in breast cancer. Biosci Rep. 2021;41(1):1–17.

33. Peng Z, Xu B, Jin F. Circular RNA hsa_circ_0000376 Participates in Tumorigenesis of Breast Cancer by Targeting miR-1285-3p. Technol Cancer Res Treat. 2020;19(155):1–10.

34. Lin Y, Fu F, Lv J, Wang M, Li Y, Zhang J, et al. Identification of potential key genes for HER-2 positive breast cancer based on bioinformatics analysis. Med (United States). 2020;99(1):1–8.

35. Zhou J, Yang R. Identification of key pathways and genes shared between Crohn’s disease and breast cancer using bioinformatics analysis. Oncol Lett. 2020;20(4):1–10.

36. Lv Q, Liu Y, Huang H, Zhu M, Wu J, Meng D. Identification of potential key genes and pathways for inflammatory breast cancer based on GEO and TCGA databases. Onco Targets Ther. 2020;13:5541–50.

37. Bai J, Luo Y, Zhang S. Microarray data analysis reveals gene expression changes in response to ionizing radiation in MCF7 human breast cancer cells. Hereditas. 2020;157(1):1–8.

38. Hao M, Liu W, Ding C, Peng X, Zhang Y, Chen H, et al. Identification of hub genes and small molecule therapeutic drugs related to breast cancer with comprehensive bioinformatics analysis. PeerJ. 2020;8.

39. Dong H, Zhang S, Wei Y, Liu C, Wang N, Zhang P, et al. Bioinformatic analysis of differential expression and core GENEs in breast cancer. 2018;11(3):1146–56.

40. Wang Y, Li H, Ma J, Fang T, Li X, Liu J, et al. Integrated Bioinformatics Data Analysis Reveals Prognostic Significance Of SIDT1 In Triple-Negative Breast Cancer. Onco Targets Ther. 2019;12:8401–10.

41. Pei J, Wang Y, Li Y. Identification of key genes controlling breast cancer stem cell characteristics via stemness indices analysis. J Transl Med. 2020;18(1):1–15.

42. Li Y, Zhou X, Liu J, Yin Y, Yuan X, Yang R, et al. Differentially expressed genes and key molecules of BRCA1/2-mutant breast cancer: Evidence from bioinformatics analyses. PeerJ. 2020;2020(1):1–20.

43. Cai Y, Mei J, Xiao Z, Xu B, Jiang X, Zhang Y, et al. Identification of five hub genes as monitoring biomarkers for breast cancer metastasis in silico. Hereditas. 2019;156:20.

44. Takeshita T, Yan L, Peng X, Kimbung S, Hatschek T, Hedenfalk IA, et al. Transcriptomic and functional pathway features were associated with survival after pathological complete response to neoadjuvant chemotherapy in breast cancer. Am J Cancer Res. 2020;10(8):2555–69.

45. Tang D, Zhao X, Zhang L, Wang Z, Wang C. Identification of hub genes to regulate breast cancer metastasis to brain by bioinformatics analyses. J Cell Biochem. 2019;120(6):9522–31.

46. Qi L, Zhou B, Chen J, Hu W, Bai R, Ye C, et al. Significant prognostic values of differentially expressed-aberrantly methylated hub genes in breast cancer. J Cancer. 2019;10(26):6618–34.

47. Wang Y, Xu H, Zhu B, Qiu Z, Lin Z. Systematic identification of the key candidate genes in breast cancer stroma. Cell Mol Biol Lett. 2018;23(1):1–15.

48. Fang E, Zhang X. Identification of breast cancer hub genes and analysis of prognostic values using integrated bioinformatics analysis. Cancer Biomarkers. 2017;21(1):169–77.

49. Lou W, Liu J, Ding B, Xu L, Fan W. Identification of chemoresistance-associated miRNAs in breast cancer. Cancer Manag Res. 2018;10:4747–57.

50. Fu Y, Zhou QZ, Zhang XL, Wang ZZ, Wang P. Identification of hub genes using co-expression network analysis in breast cancer as a tool to predict different stages. Med Sci Monit. 2019;25:8873–90.

51. Bao C, Lu Y, Chen J, Chen D, Lou W, Ding B, et al. Exploring specific prognostic biomarkers in triple-negative breast cancer. Cell Death Dis. 2019;10(11).

52. Zhong G, Lou W, Shen Q, Yu K, Zheng Y. Identification of key genes as potential biomarkers for triple-negative breast cancer using integrating genomics analysis. Mol Med Rep. 2020;21(2):557–66.

53. Bhar A, Haubrock M, Mukhopadhyay A, Maulik U, Bandyopadhyay S, Wingender E. Coexpression and coregulation analysis of time-series gene expression data in estrogen-induced breast cancer cell. Algorithms Mol Biol. 2013;8(1):1–11.

54. Wang Y, Zhang Y, Huang Q, Li C. Integrated bioinformatics analysis reveals key candidate genes and pathways in Breast cancer. Mol Med Rep. 2018;17(6):8091–100.

55. Dashti S, Taheri M, Ghafouri-Fard S. An in-silico method leads to recognition of hub genes and crucial pathways in survival of patients with breast cancer. Sci Rep. 2020;10(1):1–13.

56. Peng C, Ma W, Xia W, Zheng W. Integrated analysis of differentially expressed genes and pathways in triple-negative breast cancer. Mol Med Rep. 2017;15(3):1087–94.

57. Liu X, Ma Y, Yang W, Wu X, Jiang L, Chen X. Identification of therapeutic targets for breast cancer using biological informatics methods. Mol Med Rep. 2015;12(2):1789–95.

58. Bao S, Chen Y, Yang F, Sun C, Yang M, Li W, et al. Screening and Identification of Key Biomarkers in Acquired Lapatinib-Resistant Breast Cancer. Front Pharmacol. 2020;11(September):1–11.

59. Yin X, Wang P, Yang T, Li G, Teng X, Huang W, et al. Identification of key modules and genes associated with breast cancer prognosis using WGCNA and ceRNA network analysis. Aging (Albany NY). 2021;13(2):2519–38.

60. Zhang J, Zhou YJ, Yu ZH, Chen AX, Yu Y, Wang X, et al. Identification of core genes and clinical roles in pregnancy-associated breast cancer based on integrated analysis of different microarray profile datasets. Biosci Rep. 2019;39(6):1–12.

61. Zheng T, Wang A, Hu D, Wang Y. Molecular mechanisms of breast cancer metastasis by gene expression profile analysis. Mol Med Rep. 2017;16(4):4671–7.

62. Wang YW, Zhang W, Ma R. Bioinformatic identification of chemoresistance-associated microRNAs in breast cancer based on microarray data. Oncol Rep. 2018;39(3):1003–10.

63. Wang J, Yu H, Yili A, Gao Y, Hao L, Aisa HA, et al. Identification of hub genes and potential molecular mechanisms of chickpea isoflavones on MCF-7 breast cancer cells by integrated bioinformatics analysis. Ann Transl Med. 2020;8(4):86–86.

64. Zeng F, Fu J, Hu F, Tang Y, Fang X, Zeng F, et al. Identification of key pathways and genes in response to trastuzumab treatment in breast cancer using bioinformatics analysis. Oncotarget. 2018;9(63):32149–60.

65. Qin J, Chen YH. Molecular-level effects of eribulin and paclitaxel on breast cancer based on differential co-expression network analysis. Genet Mol Res. 2016;15(2).

66. Zhang M, Gao CE, Li WH, Yang Y, Chang L, Dong J, et al. Microarray based analysis of gene regulation by mesenchymal stem cells in breast cancer. Oncol Lett. 2017;13(4):2770–6.

67. Yuan CL, Jiang XM, Yi Y, Jian-Fei E, Zhang ND, Luo X, et al. Identification of differentially expressed lncRNAs and mRNAs in luminal-B breast cancer by RNA-sequencing. BMC Cancer. 2019;19(1):1–12.

68. Hong Z, Wang Q, Hong C, Liu M, Qiu P, Lin R, et al. Identification of seven cell cycle-related genes with unfavorable prognosis and construction of their TF-miRNA-mRNA regulatory network in breast cancer. J Cancer. 2020;12(3):740–53.

69. Alam MS, Rahaman MM, Sultana A, Wang G, Mollah MNH. Statistics and network-based approaches to identify molecular mechanisms that drive the progression of breast cancer. Comput Biol Med. 2022;145.

70. Alam MS, Sultana A, Reza MS, Amanullah M, Kabir SR, Mollah MNH. Integrated bioinformatics and statistical approaches to explore molecular biomarkers for breast cancer diagnosis, prognosis and therapies. PLoS One. 2022;17(5 5).

71. Alam MS, Sultana A, Wang G, Haque Mollah MN. Gene expression profile analysis to discover molecular signatures for early diagnosis and therapies of triple-negative breast cancer. Front Mol Biosci. 2022;9:1049741.

72. Alam MS, Sultana A, Sun H, Wu J, Guo F, Li Q, et al. Bioinformatics and network-based screening and discovery of potential molecular targets and small molecular drugs for breast cancer. Front Pharmacol. 2022;13.

73. Rodrigues R, Duarte D, Vale N. Drug Repurposing in Cancer Therapy: Influence of Patient’s Genetic Background in Breast Cancer Treatment. Int J Mol Sci. 2022;23(8).

74. Balbuena-Rebolledo I, Padilla-Martínez II, Rosales-Hernández MC, Bello M. Repurposing fda drug compounds against breast cancer by targeting egfr/her2. Pharmaceuticals. 2021;14(8).

75. Pathak Y, Camps I, Mishra A, Tripathi V. Targeting notch signaling pathway in breast cancer stem cells through drug repurposing approach. Mol Divers. 2022;

76. Jubie S, Durai U, Latha S, Ayyamperumal S, Wadhwani A, Prabha T. Repurposing of Benzimidazole Scaffolds for HER2 Positive Breast Cancer Therapy: An In-Silico Approach. Curr Drug Res Rev. 2020;13(1):73–83.

77. Khanjani F, Jafari L, Azadiyan S, Roozbehi S, Moradian C, Zahiri J, et al. Drug repositioning based on gene expression data for human HER2-positive breast cancer. Arch Biochem Biophys. 2021;712.

78. Song J, Xu Z, Cao L, Wang M, Hou Y, Li K. The discovery of new drug-target interactions for breast cancer treatment. Molecules. 2021;26(24).

79. Saha Detroja T, Detroja R, Mukherjee S, Samson AO. Identifying Hub Genes Associated with Neoadjuvant Chemotherapy Resistance in Breast Cancer and Potential Drug Repurposing for the Development of Precision Medicine. Int J Mol Sci. 2022;23(20).

80. Firoozbakht F, Rezaeian I, Rueda L, Ngom A. Computationally repurposing drugs for breast cancer subtypes using a network-based approach. BMC Bioinformatics. 2022;23(1).

81. Rui M, Cai M, Zhou Y, Zhang W, Gao L, Mi K, et al. Identification of Potential RBPJ-Specific Inhibitors for Blocking Notch Signaling in Breast Cancer Using a Drug Repurposing Strategy. Pharmaceuticals. 2022;15(5).

82. Mok K, Tsoi H, Man EP, Leung M, Chau KM, Wong L, et al. Repurposing hyperpolarization‐activated cyclic nucleotide‐gated channels as a novel therapy for breast cancer. Clin Transl Med. 2021;11(11).

83. Al-Taie Z, Hannink M, Mitchem J, Papageorgiou C, Shyu CR. Drug repositioning and subgroup discovery for precision medicine implementation in triple negative breast cancer. Cancers (Basel). 2021;13(24).

84. Brasó-Maristany F, Paré L, Chic N, Martínez-Sáez O, Pascual T, Mallafré-Larrosa M, et al. Gene expression profiles of breast cancer metastasis according to organ site. Mol Oncol. 2022;16(1):69–87.

85. Li L, Li L, Sun Q. High expression of cuproptosis-related SLC31A1 gene in relation to unfavorable outcome and deregulated immune cell infiltration in breast cancer: an analysis based on public databases. BMC Bioinformatics. 2022;23(1).

86. Ren Q, Khoo WH, Corr AP, Phan TG, Croucher PI, Stewart SA. Gene expression predicts dormant metastatic breast cancer cell phenotype. Breast Cancer Res. 2022;24(1).

87. Griguolo G, Bottosso M, Vernaci G, Miglietta F, Dieci MV, Guarneri V. Gene-expression signatures to inform neoadjuvant treatment decision in HR+/HER2− breast cancer: Available evidence and clinical implications. Cancer Treat Rev. 2022;102.

88. Prat A, Guarneri V, Pascual T, Brasó-Maristany F, Sanfeliu E, Paré L, et al. Development and validation of the new HER2DX assay for predicting pathological response and survival outcome in early-stage HER2-positive breast cancer. eBioMedicine. 2022;75.

89. Cebulski K, Nowińska K, Jablońska K, Romanowicz H, Smolarz B, Dzięgiel P, et al. Expression of Irisin/FNDC5 in Breast Cancer. Int J Mol Sci. 2022;23(7).

90. Meng Y, Ying Y, Zhang M, Zhang S, Yao Y, Li D. A comprehensive bioinformatic analysis of RanBP9 expression and its relation to prognosis in human breast cancer. Epigenomics. 2022;14(1):27–42.

91. Zhu L, Tian Q, Gao H, Wu K, Wang B, Ge G, et al. PROX1 promotes breast cancer invasion and metastasis through WNT/β-catenin pathway via interacting with hnRNPK. Int J Biol Sci. 2022;18(5):2032–46.

92. Fang Y, Liu J, Zhang Q, She C, Zheng R, Zhang R, et al. Overexpressed VDAC1 in breast cancer as a novel prognostic biomarker and correlates with immune infiltrates. World J Surg Oncol. 2022;20(1).

93. Panagopoulou M, Drosouni A, Fanidis D, Karaglani M, Balgkouranidou I, Xenidis N, et al. ENPP2 Promoter Methylation Correlates with Decreased Gene Expression in Breast Cancer: Implementation as a Liquid Biopsy Biomarker. Int J Mol Sci. 2022;23(7).

94. Yin L, Li Q, Mrdenovic S, Chu GCY, Wu BJ, Bu H, et al. KRT13 promotes stemness and drives metastasis in breast cancer through a plakoglobin/c-Myc signaling pathway. Breast Cancer Res. 2022;24(1).

95. Regua A, Papp C, Grageda A, Porter BA, Caza T, Bichindaritz I, et al. ABI1-based expression signature predicts breast cancer metastasis and survival. Mol Oncol. 2022;16(14):2632–57.

96. Grassini D, Cascardi E, Sarotto I, Annaratone L, Sapino A, Berrino E, et al. Unusual Patterns of HER2 Expression in Breast Cancer: Insights and Perspectives. Pathobiology. 2022;89(5):278–96.

97. Liu Z, Liu J, Ebrahimi B, Pratap UP, He Y, Altwegg KA, et al. SETDB1 interactions with PELP1 contributes to breast cancer endocrine therapy resistance. Breast Cancer Res. 2022;24(1).

98. Yuan K, Wu M, Lyu S, Li Y. Identification of prognostic genes for early basal-like breast cancer with weighted gene co-expression network analysis. Med (United States). 2022;101(42):E30581.

99. Chen DL, Cai JH, Wang CCN. Identification of Key Prognostic Genes of Triple Negative Breast Cancer by LASSO-Based Machine Learning and Bioinformatics Analysis. Genes (Basel). 2022;13(5).

100. Shi SC, Zhang Y, Wang T. High RRM2 expression has poor prognosis in specific types of breast cancer. PLoS One. 2022;17(3 March).
