# Supplementary material for: Exploring bacterial key genes and therapeutic agents for breast cancer among the Ghanaian female population: Insights from In Silico analyses
Source: PLoS One. 2024 Nov 25;19(11):e0312493. doi: 10.1371/journal.pone.0312493 (PMC11588272; doi:10.1371/journal.pone.0312493)
Supplement: S5 Table — (DOCX) [file pone.0312493.s006.docx]

S5 Table: Metadata of ligand molecules of breast cancer patients obtained by reviewing published articles were used in this study

|  | **Drug List** |
| --- | --- |
| Drug-Set-1 | Gallium nitrate, Cladribine, Hydroxyurea, Motexafin Gadolinium, Gemcitabine, Clofarabine, Fludarabine Phosphate, Dinaciclib, Triapine, Fludarabine, AT-7519, Alvocidib, AZD-5438, Roniciclib, TG-02, Genistein, Cordycepin, Suramin, seliciclib, RG-547, CHEMBL1236539 |
| Drug-Set-2 | Progesterone, Levonorgestrel , Estropipate, Tamoxifen, Estradiol, Danazol, Diethylstilbestrol, Desogestrel, Mestranol, Clomifene, Polyestradiol Phosphate, Fulvestrant, Norgestimate, Medroxyprogesterone, Estrone, Dienestrol, Naloxone, Tibolone, Quinestrol, Ethynodiol Diacetate, Fluoxymesterone, Estramustine, Promestriene, Estriol, Toremifene, Lasofoxifene, Etonogestrel, Trilostane, Ospemifene, Norgestrel, Allylestrenol, Raloxifene, Megestrol, Bazedoxifene, Ethinyl Estradiol, Chlorotrianisene. |
| Drug-Set-3 | Trametinib, selumetinib, and RDEA119 |
| Drug-Set-4 | NVP-BHG712, Nilotinib, GSK2126458, YM201636, TG-02, CX-5461, AP-24534 |
| Drug-Set-5 | Imatinib, regorafenib, pazopanib, teniposide, and dexrazoxane |
| Drug-Set-6 | YM201636, masitinib, SB590885, GSK1070916, GSK2126458, ZSTK474, dasatinib, fedratinib, dabrafenib, methotrexate, trametinib, tubastatin A, BIX02189, CP466722, afatinib, and belinostat |
| Drug-Set-7 | Doxorubicin, Cyclophosphamide, Everolimus, Tamoxifen, Anastrozole, Paclitaxel, Aspirin |
| Drug-Set-8 | Alfuzosin, Antrafenine, Bopindolol, Carvedilol, Doxasozin, Irinotecan, Pindolol, Prazosin, Quinacrine, Saprisartan, Sildenafil, Terazosin, Topotecan, Trimetrexate |
| Drug-Set-9 | RO4929097, Venetoclax, Conivaptan, Elbasvir, Ergotamine, Ledipasvir, Dihydroergotamine, Lomitapide, Nilotinib, Avodart, Digoxin |
| Drug-Set-10 | Benzimidazole |
| Drug-Set-11 | Vorinostat, mocetinostat, alvocidib, CGP-60474, BMS-387032, AT-7519, and curcumin |
| Drug-Set-12 | Erlotinib, caffeine, afatinib |
| Drug-Set-13 | Homoharringtonine, bortezomib, carfilzomib, anisomycin, puromycin, anisomycin, cycloheximide, puromycin, BIO-5192, BTT-3033, TC-I-15, TCS-2314, L-mimosine, SB-683698, ATN-161, BIO-1211 |
| Drug-Set-14 | Ruxolitinib, Tranilast, Rupatadine, Ribavirin, Deferiprone, Etofylline-Clofibrate, Fingolimod, ICI-185282, PF-04217903, Raloxifene, EDTA, Amiprilose, Bafilomycin A1, Dexamethasone, Dofequidar, MK-1775, TG-100801, Swainsonine, Raclopride, L-690330, Phentermine, PHA-767491, PD-173074, Lidocaine, Mibampator, PD-153035, AKT-inhibitor-1-2, Maraviroc, SDZ-NKT-343, Clomipramine |
| Drug-Set-15 | Schaftoside, fidaxomicin, acarbose |
| Drug-Set-16 | Ivabradine and paclitaxel |
| Drug-Set-17 | Digitoxin, Homoharringtonine, Sunitinib, Idarubicin, Rifampicin, Ouabain, Chenodeoxycholic acid, Pazopanib, Topotecan, Varenicline, Digoxin, Fluvastatin, Mycophenolic acid, Mitomycin, Tubocurarine, Daunorubicin, Lovastatin, Lurasidone, Vinblastine, Cerulenin, Afatinib, Gefitinib, Mianserin, Gemcitabine, Galantamine, Bosutinib, Bosutinib, Mirtazapine, Tolazamide, Sorafenib, Lapatinib, Erlotinib, Risperidone, Levetiracetam, Topotecan, Gefitinib, Cyclosporine, Asenapine, Epirubicin, Vemurafenib, Tretinoin, Sunitinib, Cabergoline, Irinotecan, Methylphenobarbital, Mebendazole, Mycophenolic acid, Iloperidone, Nilotinib, Primidon. |
