# Supplementary material for: Exploring bacterial key genes and therapeutic agents for breast cancer among the Ghanaian female population: Insights from In Silico analyses
Source: PLoS One. 2024 Nov 25;19(11):e0312493. doi: 10.1371/journal.pone.0312493 (PMC11588272; doi:10.1371/journal.pone.0312493)
Supplement: Attachment — Submitted filename: Response to Reviewers.docx [file pone.0312493.s007.docx]

**Response to the Reviewer Comments**

**Reviewer 1**

**General Comments:** Thank you for giving us the opportunity to consider your work. Specific concerns expressed during peer review were:

In this study authors identified 26 differential genera between BC and healthy groups of bacterial sequences. They suggested top 10 bKGs and bKG-guided top-ranked 10 drug molecules that could be potential treatment strategies for bacterial infections in BC patients. The manuscript seems interesting; however, some minor revision is required before it can be published in PLOS ONE.

**Response**: Thank you so much for your important comments that help us to improve the quality of the manuscript.

**Specific comments**

**Comment 1:** Authors can add statistics of breast cancer in Africa in the introduction section.

**Response**: Thank you for your suggestion. We revised the introduction section by including statistical data about breast cancer in Africa. Please see lines 55-57 in the revised manuscript.

**Comment 2**: Workfellows should be mentioned at the end of the introduction section.

**Response:** Thanks for your suggestion. We have moved the workflow at the end of the introduction section.

**Comment 3:** Throughout the paper, terms such as Breast Cancer (BC) were repeatedly written in full form; instead, use the full form only once and consistently use the abbreviation (e.g., BC) in subsequent mentions.

**Response:** Thank you for your suggestion. We have revised the manuscript accordingly.

**Comment 4:** Similarly, some other terms such as identifying bacterial key genes (bKGs) were repeatedly written in full form as well as and also check others terms.

**Response:** Thanks for your suggestion. We have revised it accordingly.

**Comment 5:** We collected 16S rRNA sequence data from the NCBI while 520 samples were BC patients and 442 were healthy controls. Avoid using “we” when writing paper or thesis; just make sentences in passive voice.

**Response:** Thank you for your suggestion. We have corrected it in the revised manuscript. Please see the lines 31-33.

**Comment 6:** we explored top 10 bKGs (mdh, pykF, gapA, zwf, pgi, tpiA, pgk, pfkA, ppsA, and pykA) through protein-protein interactions analysis as potential pathogenic factors.
Please replace the use of “we” in the sentence as well as the previous one.
ATTENTION: There are too many “we” that been used in this paper from abstract to results and discussion, thus, please recheck and replace it with better option so that the manuscript would sounds finer.

**Response:** Thank you for your suggestion. We have carefully rechecked the entire manuscript to ensure that similar changes have been made consistently.

**Comment 7:** In Figure 5, you should mention what you represent in Y-axis and X-axis like in figure 6a.

**Response:** Thank you for your suggestion. The **Figure 5** was revised according to your suggestion in the revised manuscript.

**Comment 8:** Authors protein structures were obtained from the Protein Data Bank (PDB) database, PDB IDs of the proteins used for the docking needs to be mentioned.

**Response:** Thank you for your suggestion. PDB IDs for the receptor proteins were mentioned in the revised manuscript. Please see lines 113-114 and 299-300.

**Comment 9:** Author suggests to make three-line format tables.

**Response:** Thank you for your suggestion. The tables in the revised manuscript have been updated accordingly. **Comment 10**: Go through the entire manuscript, looking for any typos and grammatical issues. Clarity of statements should be checked in several places.

**Response:** Thank you for your suggestion. We have thoroughly reviewed the manuscript and corrected all typos and grammatical issues to improve clarity throughout the revised manuscript.

**Comment 11:** Author identified 26 differential genera between BC and healthy groups. How they specifically mentioned later that the two genera Prevotella and Anaerovibria were significantly enriched among them in BC patients? In lines 32-33

**Response:** Thank you for your suggestion. We have revised the sentence in the updated manuscript. Please see lines 35-37.

**Comments from Reviewer 2**

**General Comments:** The manuscript, titled “Exploring Bacterial Key Genes and Therapeutic Targets in Breast Cancer among the Ghanaian Population: Insights from In-Silico Analyses,” describes breast cancer (BC) as a global health issue, with microbiota and epigenetic changes being significant risk factors. A study in Ghana identified bacterial key genes (bKGs) associated with BC infections and explored potential drug molecules targeting these bKGs. The study found significant bacterial discrepancies between BC and healthy groups, with Prevotella and Anaerovibria genera enriched in BC patients. The top 10 bKGs were identified as potential pathogenic factors, and drug molecules were identified using molecular docking analysis. Following are some suggestions that, in general, improve the presentation and quality of this work:

**Response**: Thank you so much for your important comments that help us to improve the quality of the manuscript.

**Specific Comments:**

**Comment 1:** The authors should revise the title of this manuscript. Please make sure that selecting a research manuscript topic with qualities like originality, relevance, specificity, feasibility, interest, impact potential, interdisciplinary relevance, ethical considerations, clarity, and contribution to knowledge significantly impacts its success.

**Response:** Thank you for your valuable suggestion. We revised the title of the manuscript in the revised manuscript.

**Comment 2**: The abstract contains excessive detail and should be rewritten to clarify the rationale for mentioning the study’s objectives in a more prominent manner and remove redundant expressions. For instance, see lines 29–31: “To do this, we collected 16S rRNA sequence data... controls.”. Please justify how you collect the sequence data. The word “”collected” seems to mismatch with this information. Please re-check the use of some words like line 31 ('discrepancies'), line 36 ('explored'), line 43 (‘valuable insight'), ‘potential treatment strategies', etc.

**Response:** Thank you for your valuable suggestion. We revised the abstract accordingly. Excessive details and redundant phrases have been eliminated. In the revised manuscript, we wrote that the sequence data were downloaded from NCBI database (please see lines: 31-33). Terms such as "discrepancies," "explored," "valuable insights," and "potential treatment strategies" have been revised for accuracy and relevance. Please see the revised abstract.

**Comment 3:** Keywords: A few of them should not be included at this level. The authors should re-check the suitability of this section and only add the prominent keywords that are significant to this work, which will help the global readers appropriately, select this work in the future.

**Response:** Thank you for your important suggestion regarding the keywords. We updated the keywords in the revised manuscript. Please see lines 48-49.

**Comment 4:** Introduction: The introduction isn’t organized and looks more like a mishmash of information, with several grammar and punctuation errors. Authors should comprehensively revise this section, as a good manuscript introduction should provide clear context, highlight the study's importance, address the specific research problem, summarize the current state of research, justify the research approach, define the study's scope and focus, be engaging and accessible, and outline the structure of the manuscript. For instance, “The World Health Organization reported 2.3 million women were diagnosed with BC in 2020, resulting in 68,500 deaths worldwide, with new cases expected to rise to 3.06 million annually by 2040” needs a proper citation. Lines 55–56, the mentioned statement has some confusion; kindly re-check and revise accordingly. Please re-check lines 65–67. Similarly, please justify why you mentioned lines 80–83. Introduction should also provide a literature review, justify the research approach, and provide a clear narrative that aligns with the study's objectives. Unfortunately, the present state of the manuscript does not address the above attributes of the introduction. What do you mean by the statement ‘valuable insight’? Avoid jargon and overly technical language for a broad audience (please revise lines 95–96). Incorporating these elements will help create a well-rounded and effective introduction that captures the reader’s interest and clearly communicates the significance and direction of the research.

**Response:** Thanks for your insightful comments regarding the introduction section. We rewrite introduction section accordingly in the revised manuscript. Please see lines 50-91.

**Comment 5:** Methodology: sub-headings 2.1 and 2.2 need to be revised with more detail and explanation. Similarly, the content described in the 2.1 sections needs more description with thorough literature. I will suggest here to further break this sub-heading accordance to work relevance (optional). What was the justification for using or selecting published host-protein and ligand molecules of BC patients as metadata for this work? Lines 114–116: “Additionally, a total of 198 drug compounds associated with BC were collected by reviewing 17 articles.” Please cite some authentic references in support of this activity. Details mentioned in lines 120–129 need to be more organized. In 2.2.2., please justify the use of Principle Coordinate Analysis (PCoA) plots. In 2.2.3., why do the mentioned statistical analyses include the Wilcoxon signed rank test, the Mann-Whitney U test, and the Kruskal-Wallis test (please justify)? Lines 155–157 need to be more organized according to work done. Please also explain in detail: “Genes corresponding to these pathways were compiled.” Sub-heading 2.2.5. also needs more clarity in mentioning docking and simulation aspects. What is the reason for mentioning sub-heading 2.2.6.? Is this really a requirement for bioinformatics-based research? Justify.

**Response**: Thank you for your valuable comments. To address the review comments, we have revised the methodology sections for greater clarity and detail. Sub-headings 2.1 and 2.2 have been expanded to include comprehensive descriptions supported by relevant literature, detailing the justification for using host-protein and ligand molecules of BC patients. Regarding the use of metadata associated with BC by the previous independent studies, we collected 198 candidate drug compounds by the review of 17 articles (Table S5) for docking analysis with the proposed bKGs-mediated proteins. Then we selected top-ranked 10 molecules as the tentative inhibitors of bKGs. Then we verify their binding ability against top-ranked host-receptors also, since bacterial protein interacts with host-proteins to develop infections. The Principal Coordinate Analysis (PCoA) plots were used to visualize beta diversity and assess scale differences in bacterial community composition. Please see lines 141-142. Actually, we used Likelihood Ratio Test (LRT) procedure to investigate differentially abundant bacterial composition between BC and control samples from the viewpoints of statistical significance. Please see lines 152-153. The other test procedure mentioned in the manuscript can also be used. However, we remove those approaches to avoid the confusion. For clarity, we rewrite the BC-causing gene selection section. We also revised the sub-heading 2.2.5 in the revised manuscript. Please see lines 154-161. We remove section 2.2.6 to the declaration part.

**Comment 6:** The attached work flow is of poor quality and visual. Authors should avoid this limited and primary-level work flow. I would suggest the authors add an appealing workflow with visuals, as shown in a variety of manuscripts, to enhance the understanding of their work. Please check the following manuscripts for a better reference.
DOI: 10.1021/acsomega.2c04871
DOI: 10.1021/acsptsci.2c00212

DOI: 10.1021/acs.jcim.0c00488

**Response**: Thank you for your suggestion. We have updated the study workflow as recommended in the revised manuscript. Please see Fig 1.

**Comment 7**: Results: The output seems satisfactory; however, the language used by the authors when describing their outcomes frequently overstates the conclusions that can be drawn based solely on computational studies lacking experimental data. Most sub-headings in these sections, along with the subsequent details, tend to overstate facts. Currently, this part of the manuscript is wordy and contains some redundancies. For instance, sub-heading 3.3 needs more improvement with relevant details. What is the purpose of beta-diversity analysis (justify)? In Table 1, please mention the inference you extracted from the LogFC value analysis (for the readers) regarding genes selection. With reference to Figure 5, a Pearson correlation analysis was conducted between the 26 differentially abundant genera and 19 metabolic pathways. Based on this fact, Pearson correlation is more sensitive to outliers, which can significantly affect the correlation coefficient since it relies on the mean and standard deviation. Whereas Spearman correlation is less sensitive to outliers as it uses ranks instead of actual data values, reducing the impact of extreme values. The author should justify the use of Pearson correlation analysis. Data are presented in figures and left to the reader to deduce their relevance and appropriateness. The details available in lines 275-278 and 280-282 need more clarity. Figures 5 and 6 need more discussion on the basis of their outcomes. Similarly, in Figure 7, the comparative discussion needs to be incorporated from the MD simulation analysis. While the manuscript is good, it lacks breadth, largely due to its verbosity and vagueness in the Results and Discussion sections. I believe a rewrite would strengthen this manuscript.

**Response**: Thank you for your important comments on the results section of our manuscript. We acknowledge the concern regarding the language used, and we revised the text to avoid overstating conclusions. We addressed the verbosity and redundancies, particularly in sub-heading 3.3, by streamlining the content and providing more precise details. Please see lines 224-228. To observe the bacterial discrepancy between BC and control samples, we performed beta-diversity and alpha-diversity analyses. We also state this justification in the revised manuscripts. Please see lines: 203-229, 215-216. Additionally, we included a clear interpretation of the Log2FC value analysis in Table 1 to aid readers in understanding gene selection criteria. Please see lines 246-248 and 258-260. Thanks for your important suggestion. In the revised manuscript, we applied Spearman rank correlation instead of Pearson correlation to minimize outlier effect. Please see lines 289-294. The details in lines 275-278 and 280-282 clarified for better understanding. We also enhanced the discussion surrounding Figures 5, 6, and 7, particularly integrating a comparative analysis from the MD simulation results. We appreciate your suggestions and believe that these revisions significantly improve the manuscript's clarity and impact.

**Comment 8:** Conclusion: In its present form, it is very simple and monotonous with the work scope. It will be better if this section is carefully revised.

**Response:** Thank you for your valuable suggestion. We revised the conclusion section accordingly. Please see lines 439-453.

**Comment 9**: Strengths and limitations: It will be better if you specifically write them in your manuscript.

**Response:** Thank you for the suggestion. We wrote strengths and limitations in the revised manuscript. Please see lines 434-437.

**Comment 10**: References: If possible, the author should provide some of the of the latest references in the introduction and discussion sections.

**Response:** Thank you for the suggestion. We updated both introduction and discussion sections adding new references.

**Comment 11**: Other: Some English grammar mistakes exist; please further check the manuscript and refine the language carefully.

**Response:** Thank you for pointing this out. We thoroughly reviewed the manuscript to correct any grammatical errors and refine the language to improve clarity and readability.
